# BOLD cardiorespiratory pulsatility in the brain: from noise to signal of interest

**DOI:** 10.3389/fnhum.2023.1327276

**Published:** 2024-01-08

**Authors:** Stefano Delli Pizzi, Francesco Gambi, Massimo Di Pietro, Massimo Caulo, Stefano L. Sensi, Antonio Ferretti

**Affiliations:** ^1^Department of Neuroscience, Imaging and Clinical Sciences, University “G. d’Annunzio” of Chieti-Pescara, Chieti, Italy; ^2^Comec Innovative SrL, Chieti, Italy; ^3^Institute for Advanced Biomedical Technologies (ITAB), “G. d’Annunzio” University, Chieti, Italy; ^4^UdA-TechLab, Research Center, University “G. d’Annunzio” of Chieti-Pescara, Chieti, Italy

**Keywords:** BOLD, fMRI, aging, pulsatility, neurodegenerative

## Abstract

Functional magnetic resonance imaging (fMRI) based on the Blood Oxygen Level Dependent (BOLD) contrast has been extensively used to map brain activity and connectivity in health and disease. Standard fMRI preprocessing includes different steps to remove confounds unrelated to neuronal activity. First, this narrative review explores how signal fluctuations due to cardiac and respiratory activity, usually considered as “physiological noise” and regressed out from fMRI time series. However, these signal components bear useful information about some mechanisms of brain functioning (e.g., glymphatic clearance) or cerebrovascular compliance in response to arterial pressure waves. Aging and chronic diseases can cause stiffening of the aorta and other main arteries, with a reduced dampening effect resulting in greater transmission of pressure impulses to the brain. Importantly, the continuous hammering of cardiac pulsations can produce local alterations of the mechanical properties of the small cerebral vessels, with a progressive deterioration that ultimately affects neuronal functionality. Second, the review emphasizes how fMRI can study the brain patterns most affected by cardiac pulsations in health and disease with high spatiotemporal resolution, offering the opportunity to identify much more specific risk markers than systemic factors based on measurements of the vascular compliance of large arteries or other global risk factors. In this regard, modern fast fMRI acquisition techniques allow a better characterization of these pulsatile signal components due to reduced aliasing effects, turning what has been traditionally considered as noise in a signal of interest that can be used to develop novel non-invasive biomarkers in different clinical contexts.

## 1 Introduction

The median age increase in worldwide population has led to an ever-increasing impact of aging-related diseases. Traditionally, functional magnetic resonance imaging (fMRI) in elderly individuals with or without medical conditions have focused on changes in brain activation patterns during sensory stimuli or execution of cognitive tasks, as well as on changes in intrinsic functional connectivity (resting state) and structural patterns (Esposito et al., 2013, 2018; Ferreira and Busatto, 2013; Groschel et al., 2013; Brier et al., 2014).

Functional magnetic resonance imaging is mainly based on the Blood Oxygen Level Dependent (BOLD) signal (Bandettini et al., 1992; Ogawa et al., 1992). The BOLD response stems from a complex interplay of changes in cerebral blood flow (CBF), cerebral blood volume (CBV), and cerebral metabolic rate of oxygen consumption (CMRO_2_) induced by neuronal activation. These physiological changes alter the local content of deoxygenated hemoglobin. Since this molecule is paramagnetic, it creates magnetic field gradients in both intravascular and extravascular space, affecting the MRI signal in T2* weighted images. Corresponding functional time series are usually acquired using echo-planar imaging (EPI) sequences with a sampling rate of around 0.5 Hz (repetition time, TR = 2 s). This low temporal resolution causes aliasing phenomena produced by heart beats which appear in the time series as fluctuations at lower frequencies that, together with respiratory-related slower signal variations, may overlap with the oscillations produced by the neuronal activity (Bianciardi et al., 2009; Birn, 2012). These cardiorespiratory fluctuations are generally perceived as noise that masks BOLD responses driven by neuronal activity. For this reason, various analysis methods have been developed to reduce or eliminate the contribution of cardiac and respiratory pulsatility from the BOLD time series. However, the possibility of acquiring the fMRI signal with a much lower TR offered by the multiband technique (Feinberg et al., 2010; Feinberg and Setsompop, 2013; Moller et al., 2013) or by ultrafast fMRI based on the magnetic resonance encephalography (MREG) approach (Hennig et al., 2021), allows not only to avoid the aliasing phenomena but also to study cardiac pulsatility in the brain. These alternative approaches allow interpreting related signals not as noise but as informative of cerebrovascular alterations and heart-brain interactions.

Moreover, recent evidence indicated that cerebral arterial pulsations could drive perivascular cerebrospinal fluid flow (CSF) and affect the glymphatic functioning of the brain (Iliff et al., 2013; Nedergaard, 2013; Kiviniemi et al., 2016; Mestre et al., 2018). These fluctuations are also of interest for research into cerebral compliance in response to arterial pressure waves (Tong and Frederick, 2014; Bianciardi et al., 2016; Atwi et al., 2019) and their relationship with paraphysiological aging and related neurodegenerative disorders (Chiarelli et al., 2017; Viessmann et al., 2019; Kim et al., 2021).

The compliance can generally be defined as the change in volume of a given tissue compartment in response to a given change in pressure and is inversely linked to the rigidity of the structures that enclose the compartment. The blood volume in the cerebral arteries changes cyclically due to the cardiac pulsations, which can therefore be considered as endogenous pressure pulses useful to study some of the mechanisms of heart-brain interaction. Compliance is an important index of the biomechanical properties of the tissues of the human body, in the healthy and pathological subjects. Aging and related diseases (i.e., hypertension, dyslipidemia, and diabetes) can cause stiffening of the aorta and other main arteries which results in greater transmission of pressure impulses in the more peripheral vessels and in particular in the cerebral ones. These repeated impulses can in turn produce local alterations of the mechanical properties of the cerebral vessels with an increased risk of stenosis, formation or detachment of plaques, as well as a substantial deterioration that is reflected in the long term also on the neuronal functionality (Baumbach et al., 1991; Boutouyrie et al., 1999; Gupta et al., 2012; Makedonov et al., 2013). Indeed, there is increased evidence that vascular issues play a major role not only in brain pathologies directly involving blood vessels (e.g., stroke) but in neurodegenerative disease as well, with a potential link between arterial phenotypes in mid-life and increased risk of dementia (Poels et al., 2007; Mitchell et al., 2011; Pase et al., 2012; Singer et al., 2013; van Sloten et al., 2015; Lin et al., 2018).

The most employed non-invasive marker of systemic compliance is the carotid-femoral pulse wave velocity (cfPWV), determined from the time taken by the arterial pulse to travel from the carotid to the femoral artery. Large arteries in the brain are also accessible with non-invasive methods suitable to estimate vessel compliance, such as Transcranial Doppler Ultrasound (TCD). In this case, a carotid pulsatility index is usually defined as the difference between the peak systolic flow and minimum diastolic flow velocity, normalized to the mean velocity recorded throughout the cardiac cycle.

However, high spatio/temporal resolution fMRI studies of the brain patterns most affected by cardiac pulsations in health and disease may offer the opportunity to identify much more specific risk markers than systemic factors based on measurements of the vascular compliance of large arteries or other global risk factors related to hypertension, dyslipidemia, diabetes, etc. This paper reviews the main studies on this emerging fMRI application in major neurological and psychiatric disorders.

## 2 Neurological studies

### 2.1 Aging and dementia

In the past 15 years, research on aging and dementia showed a relationship between systemic vascular factors and brain function. The nature of this association was extensively examined by measuring the vascular stiffness of large arteries (Poels et al., 2007; Mitchell et al., 2011; Pase et al., 2012; Singer et al., 2013; Lin et al., 2014; van Sloten et al., 2015). Pulsatile hemodynamic changes, measured through cfPWV, precedes the onset of hypertension, suggesting that arterial stiffness serves as a powerful predictor of cognitive decline, rather than hypertension alone (Kaess et al., 2012; Hajjar et al., 2016). However, the causality direction between aortic stiffening and hypertension is complex, potentially involving a bidirectional relationship (Keehn et al., 2023). Interestingly, higher cfPWV was linked to increased white matter hyperintensity (WMH) volume, which serves as a marker for cerebral injury (Keehn et al., 2023). Elevated values of the carotid pulsatility index, pulse pressure, and cfPWV have each been linked to an increased risk of silent subcortical infarcts, which are defined as infarcts observed on brain CT or MRI without any corresponding stroke episode (Raghavan et al., 2021). The Carotid pulsatility index was associated with lower whole-brain volumes of the gray matter (GM) and white matter (WM), which are in turn indicative of overall brain atrophy. Carotid pulsatility index was also correlated with the decline in a broad range of cognitive domains, including memory scores, processing speed, and executive function. Alteration of the cfPWV and carotid pulse pressure were selectively associated with memory impairment (Mitchell et al., 2011). In addition, cfPWV was associated with a greater risk of conversion to dementia in patients with mild cognitive impairment (MCI) (Rouch et al., 2018). In contrast, intima-media thickness, carotid plaques, or carotid artery diameter did not predict conversion to dementia. These results thereby suggest that arterial stiffness is a more specific biomarker to identify MCI patients at higher risk of dementia. Similar findings were observed by assessing pulsatility with more localized approaches targeting large cerebral arteries, e.g., using phase contrast MRI or TCD, rather than systemic indices such as cfPWV (Shi et al., 2018). In one study, the cerebral arterial stiffness assessed by the TCD ultrasound pulsatility index was correlated with the WMH lesion volume (Kidwell et al., 2001). In another study using phase contrast MRI, cerebral small vessel disease (SVD) was associated with increased intracranial pulsatility rather than with low global cerebral blood flow (Shi et al., 2020). However, both TCD and phase contrast MRI have limited spatial resolution and can usually target only large cerebral vessels. In this regard, fMRI techniques are expected to provide a more spatially refined assessment of brain pulsatility, potentially offering more specific markers to study cerebrovascular compliance or for predicting cerebrovascular/neurodegenerative disease (Dagli et al., 1999; Tong and Frederick, 2014; Bianciardi et al., 2016; Viessmann et al., 2017). For example, BOLD pulsatility in healthy adolescents was reduced after aerobic exercise in gray/white matter and in particular in the left insula, a region that regulates autonomic signals (Theyers et al., 2019). In the same vein a dynamic modulation of pulsation amplitudes during a breath hold challenge was observed, with a significant increase of pulsatility in key areas of the respiratory control network (Raitamaa et al., 2019). Makedonov et al. (2013) were the first to investigate BOLD pulsatility changes related to normal and pathological aging. Using a fast fMRI acquisition (TR = 250 ms), they achieved unaliased sampling of cardiac pulsatility in the BOLD time-series acquired in healthy young/elderly subjects and a group of patients with SVD. When investigating normal-appearing white matter (NAWM) regions, cardiac pulsatility significantly increased with age. A further significant increase was observed in SVD patients compared to healthy elderly, consistently with the notion that age-related increase in systemic arterial stiffness causes increased transmission of cardiac pressure pulses to distant organs and that pathological conditions such as SVD can contribute significantly to exacerbate this effect. However, within the SVD group, cardiac pulsatility was observed to be higher in normal-appearing white matter (NAWM) regions than in regions with white matter hyperintensity (WMH). WMH is characterized by a local hypointense signal in FLAIR MR images, indicative of leukoaraiosis, and is associated with cognitive impairment as well as with triples the risk of stroke and doubles the risk of dementia (Makedonov et al., 2013). This apparently contrasting result can be explained by observing that histological analysis showed a decreased concentration of afferent vessels within WMH regions compared to NAWM (Brown et al., 2007), which in turn could contribute to decreased pulsatility. Furthermore, the thickening of vein walls in SVD due to the deposition of collagen (Moody et al., 1995) could decrease the level of cardiac pulsatility in the affected regions. An MRI/histology study in AD revealed that collagenosis in large veins significantly predicted the WMH lesion volume (Gao et al., 2012). A more recent study performed with high-temporal resolution rs-fMRI (TR = 328 ms) also showed an age-related spatial increase of cardiac BOLD pulsatility in WM (Viessmann et al., 2019). Taken together, these findings suggest that assessment of cardiac pulsatility in the WM could characterize age modifications and SVD abnormalities that are not addressed by structural MRI. Notably, conventional fMRI data also showed that physiological fluctuations in the brain (which include an aliased contribution of cardiac pulsatility for TR > 1 s) are increased and associated with cognitive decline in AD (Makedonov et al., 2016; Scarapicchia et al., 2018).

Moreover, recent ultrafast (TR = 100 ms) fMRI reported waves of reversed impulse propagation in patients, specifically in mesiotemporal and periventricular structures (Rajna et al., 2021) suggesting increased stiffness in more distal brain vasculature, possibly due to amyloid-β and tau accumulation within the perivascular space (Palmqvist et al., 2017). Consequently, the resulting narrowing of perivascular and intravascular spaces, associated with elevated amyloid-β and tau filaments, may impede the glymphatic mechanism driven by cardiovascular arterial pulsations, ultimately leading to progressive brain function impairment.

### 2.2 Parkinson’s disease

Parkinson’s disease (PD) is a progressive age-related neurodegenerative disorder characterized by motor symptoms, including tremor, rigidity, bradykinesia, postural instability/gait difficulty (PIGD), as well as cognitive impairment (Poewe et al., 2017). Cerebral small vessel disease, manifesting as white matter hyperintensity (WMH), is associated with motor deficits (Baezner et al., 2008; Buchman et al., 2013) and cognitive impairment, and it can contribute to the severity of PD symptoms (Bohnen and Albin, 2011). The BOLD signal in WM provides physiological information that is unrelated to neuronal activity and can therefore reflect features related to small blood vessels. Shirzadi et al. (2018), testing the correlation of BOLD pulsatility in WM with vascular issues (e.g., SVD) in PD patients, revealed a significant relationship of signal fluctuation with WMH volumes, systemic vascular risk burden, microvascular damage, and striatal binding ratio of the dopamine transporter. Brain pulsatility was also associated with lower motor performance, increased postural instability, and gait difficulties. By combining fMRI and diffusion imaging metrics, Shirzadi et al. (2018) suggested that microvascular pulsatility contributes to motor dysfunction through damage to small vessels in subcortical regions and the disruption of small vessel function within WM. The latter phenomenon is critical in PD, as it leads to the disconnection of the corticostriatal-thalamocortical pathways and interhemispheric connections, both of which are recognized contributors to motor dysfunction (Bohnen and Albin, 2011). This vulnerability to pulsatility is particularly pronounced in deep brain structures like WM and the basal ganglia, as they are supplied by short, penetrating arterioles with limited ability to dampen high-pressure cardiac pulses. However, the study by Shirzadi et al. (2018) is limited by a TR of 2,400 ms during data acquisition, which was too long to clearly distinguish cardiac pulsatility from other sources of signal fluctuation, such as respiration, without simultaneous cardiorespiratory tracing. Nevertheless, these findings underscore that BOLD physiological noise can serve as an additional neuroimaging measure of PD symptoms, especially those related to small vessel alterations.

### 2.3 Epilepsy

Resting-state fMRI data acquired during the inter-ictal period have shown altered functional connectivity in individuals with epilepsy (Mankinen et al., 2012; Wurina et al., 2012; Constable et al., 2013). Recent investigations have delved into BOLD signal “noise” characteristics in epilepsy, using fast fMRI with MREG acquisition. Kananen et al. (2018, 2020) revealed heightened respiratory signal fluctuations in drug-resistant epilepsy patients. These changes were observed in brainstem areas controlling the autonomic system, spanning frequencies in the respiration/parasympathetic range (0.12–0.4 Hz) and very low-frequency range (< 0.01 Hz). Importantly, these alterations remained significant even after adjusting for head motion. Furthermore, they were confirmed in preliminary findings from newly diagnosed drug-naïve epilepsy patients, ruling out a pharmacologically induced effect (Kananen et al., 2018). External measurements of cardiorespiratory activity and electroencephalography data couldn’t explain these observations. These physiological pulsation alterations were observed even in patients without mesial temporal sclerosis, often linked with drug-resistant epilepsy (Jutila et al., 2002; Lapalme-Remis and Cascino, 2016). The absence of aquaporin AQP4 channels in mesial temporal sclerosis regions, leading to impaired clearance mechanisms, may underlie these changes. This lack of clearance can alter extracellular electrolyte concentrations, increasing neuronal susceptibility to seizures (Eid et al., 2005; Marchi et al., 2007; Lundgaard et al., 2017). Exploring intrinsic fMRI physiological signal fluctuations emerges as a novel diagnostic tool for drug-resistant epilepsy, potentially detecting early glymphatic mechanism changes preceding visible mesial temporal sclerosis signs. In a recent study involving medicated and drug-naïve patients with focal epilepsy, Kananen et al. (2022) demonstrated increased synchronicity of respiratory brain pulsations, especially in periventricular, frontal, and mid-temporal regions, compared to controls. This respiratory brain synchronicity correlated positively with seizure frequency and accurately distinguished controls from medicated patients. These findings open new avenues for understanding and diagnosing epilepsy-related changes in brain pulsatility.

## 3 Psychiatric studies

The relationship between fMRI physiological noise and psychiatric disorders remains relatively underexplored. Changes in physiological noise and axonal disruption were observed in the WM of schizophrenic patients compared to normal controls (Cheng et al., 2015). Despite some overlaps, the regions showing voxel-wise temporal signal-to-noise ratio are different from those for fractional anisotropy derived from diffusion tensor imaging, suggesting a dissociation of microstructural and functional abnormalities in WM. Moreover, by employing the coefficient of variation of the BOLD signal as a proxy for physiological brain pulsatility, it has been highlighted that the patients with psychotic-like symptoms had higher mean brain pulsatility in CSF and WM compared to those without such symptoms (Saarinen A. I. L. et al., 2020). Voxel-wise analysis further revealed increased pulsatility in periventricular WM, basal ganglia, cerebellum, and cortical regions, including middle and frontal insula, primary sensorimotor and lateral paracingulate cortices, middle and frontal insula. Notably, also in this case the findings were not influenced by head motion. In addition to investigating brain pulsatility, a meta-analysis of previous fMRI and voxel-based morphometry studies in schizophrenia patients was conducted in search of affected areas using more traditional analytical approaches. Here again, only a modest overlap (approximately 6%) emerged between regions highlighted by brain pulsatility and those identified through standard functional or structural MRI analyses by Saarinen A. I. L. et al. (2020). These authors also explored potential associations between familial risk for psychosis and polygenic risk scores for schizophrenia with the coefficient of variation of the BOLD signal in the brain (Saarinen A. et al., 2020). However, when considering age, sex, and motion in their analysis, they found no significant correlation between familial risk or polygenic risk scores and brain pulsatility in cerebrospinal fluid, WM or GM. The authors propose that individuals genetically predisposed to psychosis may exhibit heightened physiological brain fluctuation, but compensatory mechanisms or underlying processes help regulate this fluctuation until the onset of neuropsychiatric diseases. Consequently, changes in brain pulsatility may only become evident after the manifestation of these disorders.

## 4 Pharmacological studies

Few studies tried to determine the effect of drugs on the transmission of cardiac arterial pulsatility to the brain. Using multiband fMRI (TR = 0.43 s) and non-invasive simultaneous continuous recording of brachial blood pressure, Webb and Rothwell (2016) investigated brain cardiac pulsatility in healthy adults after 1 week of daily intake of amlodipine (10 mg) or propranolol-LA (160 mg) in a cross-over design, with a 2-week washout. A stronger association between peripheral cardiac cycle waveform and BOLD pulsatility in GM was observed for propranolol, suggesting a reduced dampening of cardiac pressure waves reaching the brain with respect to amlodipine. Intriguingly, although the generalization of healthy subjects’ results to patient populations is not straightforward, these findings could potentially explain the differences between the 2 drugs in stroke risk mitigation (i.e., calcium channel blockers are more effective than beta blocker) despite their similar effects on systemic blood pressure (Webb et al., 2010; Webb and Rothwell, 2014, 2016). Further clinical trials are currently in progress to assess the effect of sildenafil vs. cilostazol on cerebral pulsatility and cerebrovascular reactivity and their potential benefits to slow the progression of SVD (Webb et al., 2021).

## 5 Discussion

The physiological noise in fMRI time courses originating from cardiorespiratory activity can be transformed into a noteworthy signal of interest, leading to the development of novel non-invasive biomarkers applicable in various clinical contexts, including neurodegenerative, epileptic, psychiatric, and PD. Specifically, alterations in pulsatility within pathological brain tissue due to upstream or local vessel stiffening can be investigated with high spatiotemporal resolution using clinical scanners. The consistent impact of cardiac pulsations on the small vessels in the brain has been proposed as a significant mechanism contributing to vascular changes and an important factor in waste product elimination. The utilization of ultrafast fMRI techniques, increasingly available, holds promise for exploring these applications in larger patient populations. Such studies can contribute to evaluating treatment response and advancing the development of new therapeutic strategies.

## Author contributions

SD: Conceptualization, Writing—original draft. FG: Writing—review and editing. MD: Writing—review and editing. MC: Writing—review and editing. SS: Writing—review and editing. AF: Conceptualization, Funding acquisition, Methodology, Supervision, Writing—original draft.
